# Characterization of the Role of Tumor Necrosis Factor Apoptosis Inducing Ligand (TRAIL) in Spermatogenesis through the Evaluation of Trail Gene-Deficient Mice

**DOI:** 10.1371/journal.pone.0093926

**Published:** 2014-04-15

**Authors:** Yi-Chen Lin, John H. Richburg

**Affiliations:** Division of Pharmacology and Toxicology; Center for Molecular and Cellular Toxicology, College of Pharmacy; The University of Texas at Austin, Austin, Texas, United States of America; The University of Texas MD Anderson Cancer Center, United States of America

## Abstract

TRAIL (TNFSF10/Apo2L) is a member of the tumor necrosis factor (TNF) superfamily of proteins and is expressed in human and rodent testis. Although the functional role of TRAIL in spermatogenesis is not known, TRAIL is recognized to induce apoptosis via binding to its cognate receptors; DR4 (TRAIL-R1/TNFRSF10A) and DR5 (TRAIL-R2/TNFRSF10B). Here, we utilize *Trail* gene-deficient (*Trail* ^−/−^) mice to evaluate the role of TRAIL in spermatogenesis by measuring testis weight, germ cell apoptosis, and spermatid head count at postnatal day (PND) 28 (pubertal) and PND 56 (adult). *Trail^−/−^* mice have significantly reduced testis to body weight ratios as compared to wild-type C57BL/6J at both ages. Also, *Trail* ^−/−^ mice (PND 28) show a dramatic increase in basal germ cell apoptotic index (AI, 16.77) as compared to C57BL/6J (3.5). In the testis of adult C57BL/6J mice, the AI was lower than in PND 28 C57BL/6J mice (2.2). However, in adult *Trail* ^−/−^ mice, the AI was still higher than that of controls (9.0); indicating a relative high incidence of germ cell apoptosis. Expression of cleaved caspase-8 (CC8) and cleaved caspase-9 (CC9) (markers of the extrinsic and intrinsic apoptotic pathway, respectively) revealed a two-fold increase in the activity of both pathways in adult *Trail* ^−/−^ mice compared to C57BL/6J. Spermatid head counts in adult *Trail* ^−/−^ mice were dramatically reduced by 54% compared to C57BL/6J, indicating these animals suffer a marked decline in the production of mature spermatozoa. Taken together, these findings indicate that TRAIL is an important signaling molecule for maintaining germ cell homeostasis and functional spermatogenesis in the testis.

## Introduction

Spermatogenesis is a finely tuned process where spermatozoa are formed from spermatogonia in the testis. During spermatogenesis, germ cell apoptosis serves two important roles; removing damaged germ cells, and limiting the number of developing germ cells to match the testes supportive capacity [1], [2]. A higher apoptotic rate is also observed in pubertal animals during the initial establishment of spermatogenesis, also called the first wave spermatogenesis [3], likely as mechanism to limit this population during this period of rapid germ cell production.

Tumor necrosis factor (TNF) related to apoptosis-inducing ligand (TRAIL/Apo2L/TNFSF10) is a member of the TNF super family of proteins involved in cancer development and autoimmune diseases [4], [5], and is expressed in human and rodent testis, including germ cells, Sertoli cells, and Leydig cells [6], [7], [8], [9]. TRAIL is a Type II transmembrane protein that binds to either of two receptors in humans, DR4 (TRAIL-R1/TNFRSF10A) and DR5 (TRAIL-R2/TNFRSF10B) [10], [11]. In mice, there is only one TRAIL death receptor, TRAIL-R (MK/mDR5), and it is homologous to both human death receptors [12]. The manner by which TRAIL initiates the extrinsic apoptotic signaling pathway is well characterized. Briefly, when TRAIL binds to DR4/5 to promote its recruitment of FADD (Fas-associated protein with death domain), this allows for the recruitment and activation of the initiator caspase-8 in a complex known as the death-inducing signaling complex (DISC). Caspase-8 enzyme activation cleaves other downstream effector caspase family members to ultimately lead to the apoptotic elimination of the cell [13], [14]. Another means to induce cell apoptosis is through the intrinsic apoptotic signaling pathway. This pathway is triggered by the release of cytochrome *c* from mitochondria and its binding to monomeric apoptotic protease activating factor-1 (APAF-1) to form the apoptosome complex. The formation of this complex results in the cleavage and activation of the initiator caspase-9. The cleavage and activation of executioner caspases are similar to that seen with the extrinsic pathway [15], [16].

Recent studies have focused on the potential application of TRAIL in autoimmune diseases, anti-cancer therapy [4], [5], and Type I diabetes [17]; although, the role of TRAIL in the immune system and its functional mechanisms are still controversial [18]. Previously, we showed that the combined addition of recombinant TRAIL and anti-DR5 antibody (MD5) enhanced the ability of TRAIL to cluster and activate DR5 resulting in significant increases in apoptosis in the GC2spd (ts) germ cell line, thereby implicating a likely role for TRAIL in the modulation of germ cell apoptosis in the testes *in vivo* [19]. Even though death ligands have been implicated in the control of germ cell apoptosis [20], [21], [22], few studies have focused on the role of the TRAIL-induced signaling system in regulating apoptosis during spermatogenesis in the testis.

The aim of this study was to elucidate the role of TRAIL in the regulation of the first wave of spermatogenesis during pubertal (postnatal day 28, PND 28) and of spermatogenesis in mature aged mice (PND 56). Here, we use TRAIL gene-deficient mice to examine the importance of the TRAIL signaling system in spermatogenesis and its ability to induce apoptosis in the testis during these two developmental periods.

## Results

### *Trail^−/−^* Mice have Significantly Reduced Testis to Body Weight Ratios

The testis and body weights of *Trail* gene-deficient (*Trail^−/−^*) mice and the wild type C57Bl/6J mice at both PND 28 and 56 are shown in Table 1. *Trail^−/−^* mice had significantly smaller testes than C57BL/6J mice at both PND 28 (Table 1A, 38±1.8 mg and 43±1 mg) and PND 56 (Table 1B, 77±0.7 mg and 86±1.5 mg). There was no significant difference in body weight between *Trail^−/−^* and wild type mice at either PND 28 (Table 1A, 13.90±0.47 g and 14.51±0.37 g) or PND 56 (Table 1B, 23.41±0.37 g and 23.70±0.43 g). Accordingly, *Trail^−/−^* mice had lower testis to body weight ratios (mg/g) than wild type mice, both at PND 28 (Table 1A, 2.69±0.07 and 2.98±0.04) and PND 56 (Table 1B, 3.30±0.03 and 3.64±0.05).

**Table 1:** Body weights and testis weights of *wild type C57BL/6J and Trail* ^−/−^ mice.

A. Comparison of C57BL/6J wild type and *Trail* ^−/−^ testis to body weight ratios at PND 28				
Genotype	n	Body weight (g)	Testis weight (mg)	TW/BW ratio (mg/g)
C57BL/6J	31	14.51±0.37	43±1	2.98±0.04
*Trail^−/−^*	18	13.90±0.47	38±1.8*	2.69±0.07***
**B. Comparison of C57BL/6J wild type and ** ***Trail*** **^−/−^ testis to body weight ratios at PND 56**				
Genotype	n	Body weight (g)	Testis weight (mg)	TW/BW ratio (mg/g)
C57BL/6J	30	23.70±0.43	86±1.5	3.64±0.05
*Trail^−/−^*	21	23.41±0.37	77±0.7***	3.30±0.03***

Values represent the mean ± SEM with an asterisk identifying a significant difference from control (*p<0.05 and ***p<0.001, Student’s *t*-test).

### The Total Number of Mature Spermatids is Lower in *Trail^−/−^* Adult Mice

**Figure 1:**
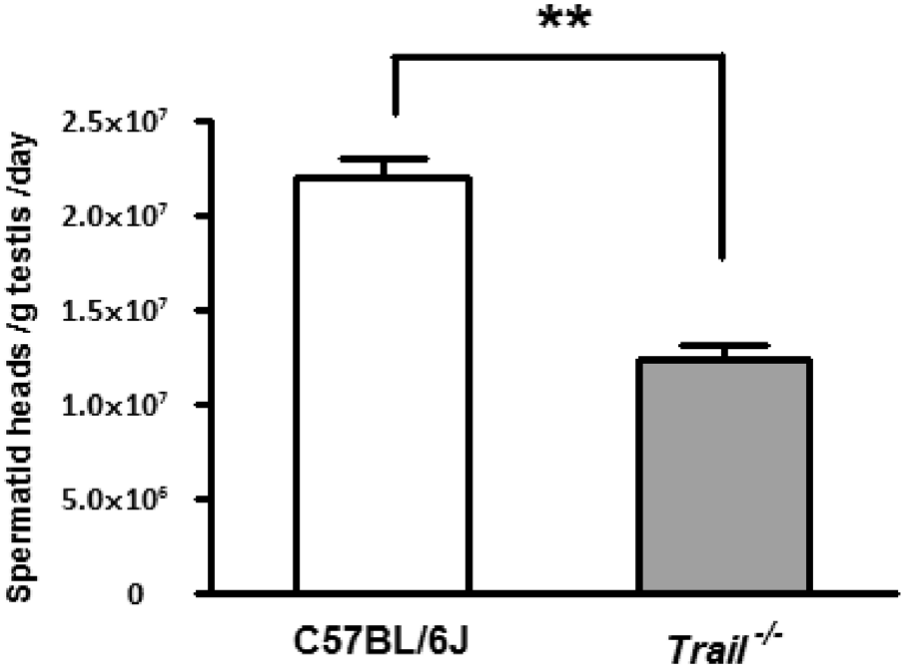
Spermatid head counts of C57BL/6J and *Trail^−/−^* mice at PND 56. The total number of mature spermatid heads were counted from 11 C57BL/6J and 8 *Trail^−/−^* mice at PND 56. *Trail^−/−^* mice have significantly lower spermatid head counts than C57BL/6J mice. Values represent the mean ± SEM with asterisks identifying a significant difference from control (**p<0.01, Student’s *t*-test).

The total number of mature spermatid heads produced per gram/testis/day was quantified in adult mice to determine if decreases in testis weight can be explained by decreases in sperm production. Wild type C57BL/6J mice had an average of [2.28±0.096]×10^7^ spermatid heads per gram/testes/day, while *Trail^−/−^* mice showed a significantly decreased number of spermatid heads ([1.24±0.08]×10^7^, Fig. 1). Compared to C57BL/6J mice, *Trail^−/−^* mice had a 54% decrease in the production of mature spermatids.

### *Trail^−/−^* Mice have Higher Rates of Germ Cell Apoptosis at Pubertal and Adult Ages

**Figure 2:**
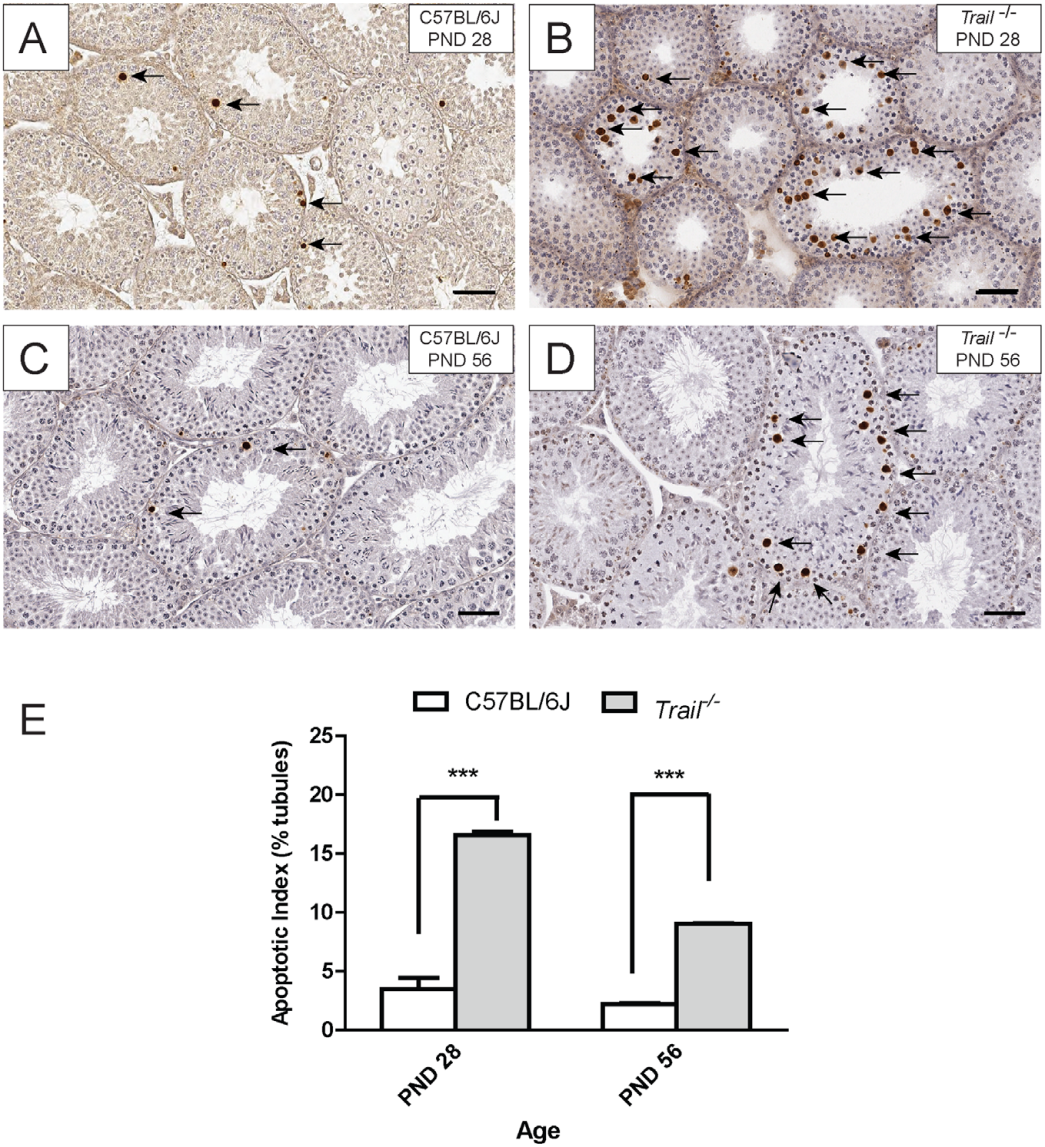
Testicular apoptotic index (AI) of C57BL/6J and *Trail^−/−^* mice. Gem cell apoptosis was identified at PND28 and PND 56 by TUNEL assay. The AI was calculated by the number of round tubules with more than 3 TUNEL-positive germ cells. The black arrows indicate selected spermatocytes undergoing apoptosis. Values represent the mean ± SEM with asterisks identifying a significant difference from control (***p<0.001, Student’s *t*-test).

TUNEL analysis was performed to determine the rate of germ cell apoptosis in *Trail^−/−^* and C57BL/6J mice at PND 28 and PND 56 (Fig. 2A–D). In C57BL/6J mice, the apoptotic index (AI) was 3.50±0.98 at PND 28 and 2.21±0.07 at PND 56, and the AI of *Trail^−/−^* mice was 16.57±0.31 at PND 28 and 9.045±0.06 at PND 56 (Fig. 2E). Comparatively, *Trail^−/−^* mice displayed a higher AI than C57BL/6J mice at both PND 28 and PND 56 (4.7 and 4.1 fold, respectively). These results indicate that *Trail^−/−^* mice have a higher basal level of germ cell apoptosis at both the pubertal and adult ages. This elevated level of apoptosis likely underlies the reduction of spermatid head counts in the adult testis.

### *Trail* ^−/−^ Mice Exhibit a Delay of in Spermatogenesis and Alterations in Meiosis at the Pubertal Age

**Figure 3:**
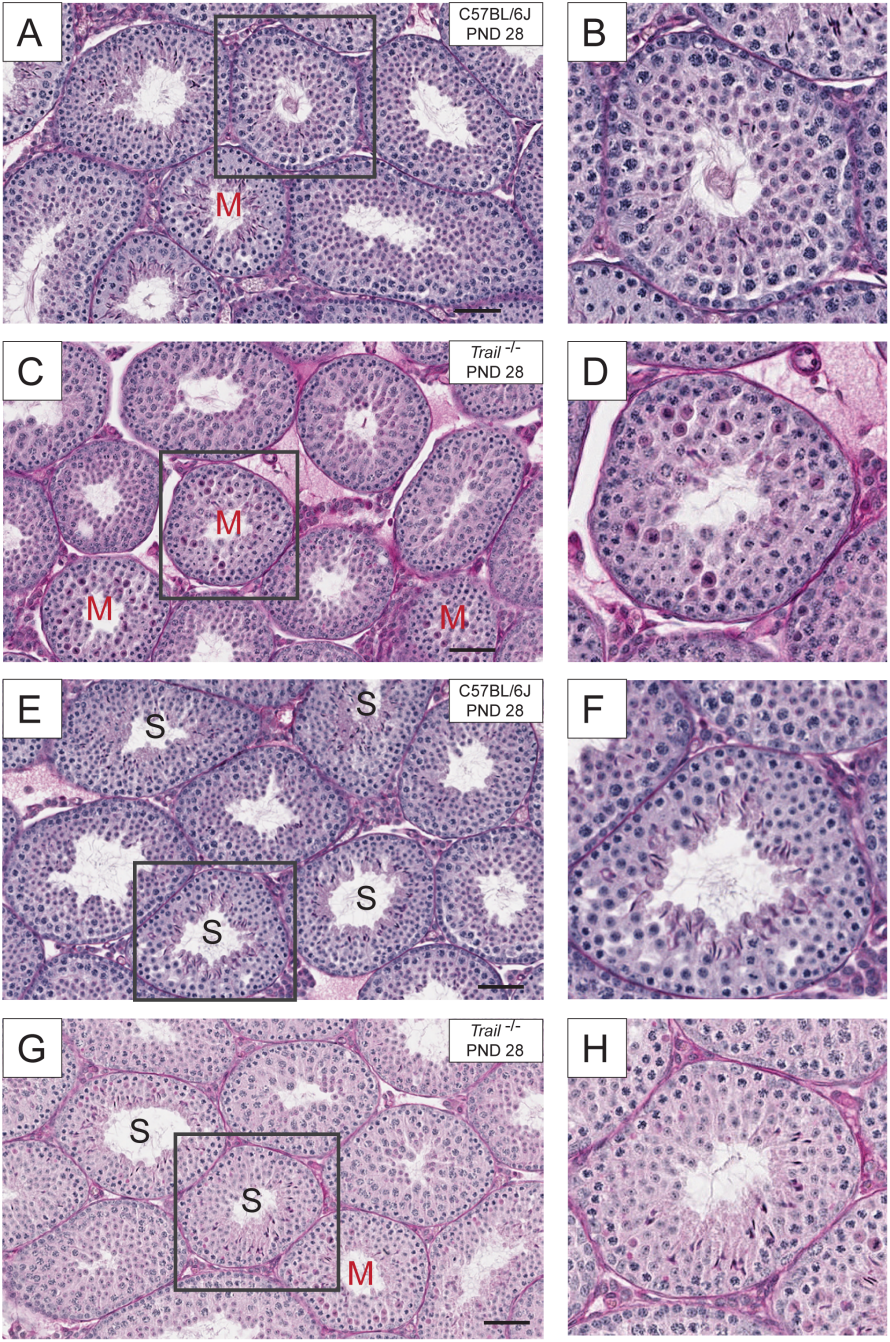
Histological evaluation of the testis of wild type C57BL/6J and *Trail^−/−^* mice at PND 28. Testicular cross sections (5 µm) from paraffin embedded tissue were examined by PAS-H (periodic acid-Schiff-hematoxylin) staining. (A) and (E) are from wild type mice, while (C) and (G) are from *Trail^−/−^* mice. The square box in each section indicates the area that is magnified in the right panel. (B, D, F, and H). *Trail^−/−^* mice appear to have more tubules containing meiotic figures (denoted with an “M”) and fewer tubules with mature spermatid heads (G, denoted with an “S”) than the wild type. The bar represents 50 µm.

**Figure 4:**
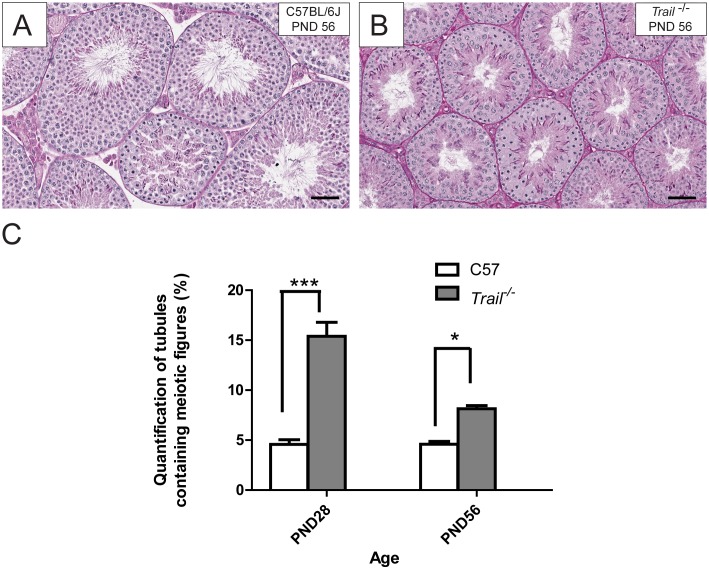
Histological evaluation of the testis of wild type C57BL/6J and *Trail^−/−^* mice at PND 56. Testicular cross sections (5 µm) from paraffin embedded tissue were examined using PAS-H staining. (A) is from wild type a C57BL/6J mouse, and (B) is from a *Trail^−/−^* mice. At PND 56, *Trail^−/−^* mice show more meiotic figures (B) than wild type C57BL/6J mice (A). (C) Quantification of tubules with meiotic figures in wild type C57BL/6J and *Trail^−/−^* mice at PND 28 and PND 56. Values represent the mean ±SEM with an asterisk identifying a significant difference from control (*p<0.05 and ***p<0.001, Student’s *t*-test). The bar represents 50 µm.

Testicular cross sections were stained using PAS-H for the histological evaluation of pubertal mice (Fig. 3 and 4). At the pubertal age, C57/BL6J mice had an average of 4.58±0.46% tubules containing meiotic germ cells (Fig. 4C), while in *Trail^−/−^* mice these events were significantly increased (15.39±1.41%, Fig. 4C). *Trail^−/−^* mice had a 3.4 fold higher incidence of meiotic figures at the pubertal age. At the adult age, *Trail^−/−^* mice had a relatively higher number of tubules containing meiotic figures (8.13±0.32%) compared to C57/BL6J mice (4.59±0.45%). Even when compared to C57/BL6J, *Trail^−/−^* mice had a greater incidence of meiotic figures (1.47 fold). These results indicate that an alteration in meiosis of germ cells occurred in *Trail^−/−^* mice. Also, the formation of mature spermatid heads at PND 28 can act as an indicator of normal physiology. Although some tubules in the wild type mice were near completion of the first wave of spermatogenesis and contained elongated spermatids in the tubules in the *Trail^−/−^* mice (Fig. 3E–F), appeared to be developmentally delayed. When comparing the tubules at the same stage, mature spermatid subtypes were present in C57/BL6J mice, but lacking in *Trail^−/−^* mice (Fig. 3G–H).

### *Trail* ^−/−^ Mice Testis Show Genotype Specific Changes in Intrinsic Apoptotic Proteins, but not in Extrinsic Apoptotic Proteins

Since TUNEL analysis revealed that the basal level of germ cell apoptosis is higher in *Trail* ^−/−^ mice than in C57BL/6J wild type mice, it is possible that either another death receptor/ligand, such as FasL, is induced or that the intrinsic ‘mitochondrial’ apoptotic signaling system has become activated. To gain insights into the participation of either the extrinsic or intrinsic signaling systems in mediating the high observed basal germ cell apoptosis in the *Trail^−/−^* testis, characteristic proteins involved in the extrinsic and intrinsic apoptotic pathways were examined by immunohistochemistry.

**Figure 5:**
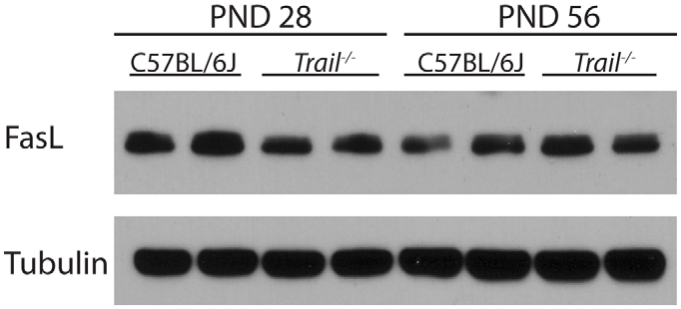
Western blot analysis of FasL expression in wild type C57BL/6J and *Trail^−/−^* mice at PND28 and PND 56. Total protein from two sets of PND 28 and PND 56 whole testis tissue were analyzed using primary antibodies against FasL. Tubulin was used as a loading control. Total cellular protein expression from whole testis homogenates at PND 28 and PND 56 were detected using primary antibodies against FasL. α-tubulin was the loading control. Values represent the mean ± SEM with an asterisk identifying a significant difference from control (*p<0.05, Student’s *t*-test).

Western blot analyses of FasL, another death ligand previously shown to cause germ cell apoptosis [20], was performed to assess if *Trail^−/−^* mice have a compensatory increase in this death receptor, which would accounts for the higher basal levels of apoptosis seen in the testis of PND 28 and 56 mice. In contrast to the induction of TRAIL observed in the testis of FasL gene deficient mice that we previously described [20], no significant differences in the expression of FasL were observed between the C57/BL6J and *Trail^−/−^* mice at either PND 28 and PND 56 (Fig. 5).

**Figure 6:**
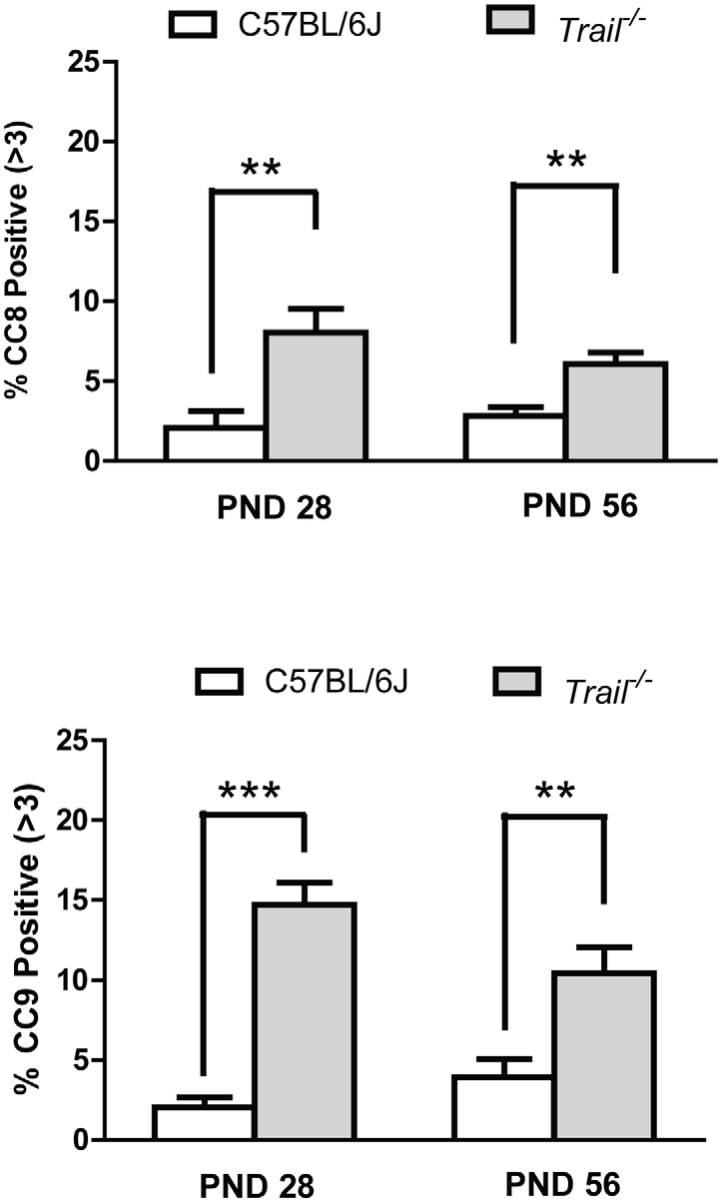
Immunohistochemical analysis of intrinsic and extrinsic apoptosis in wild type C57BL/6J and *Trail^−/−^* mice at PND 28 and PND 56. (A) Cleaved caspase-8 (CC8) and (B) cleaved caspase-9 (CC9) values were calculated as the percentage of tubules containing more than 3 CC8 or CC9 positive cells. *Trail^−/−^* mice have higher intrinsic and extrinsic apoptosis signaling at PND 28 and PND 56 than wild type mice. Values represent the mean ± SEM with an asterisk identifying a significant difference from control (**p<0.01, Student’s *t*-test).

Death receptor-mediated signaling activation was also evaluated through the detection of the cleaved form of caspase-8 (CC8, Fig. 6A), the prototypical ‘initiator’ caspase for the extrinsic signaling pathway. *Trail* ^−/−^ mice were observed to have a greater number of CC8 positive tubules than C57BL6J at both PND 28 and PND56. At PND 28, 8.04% of the tubules in *Trail* ^−/−^ mice were CC8 positive, whereas C57BL/6J mice only had 2.07% positive tubules. By PND 56, the percentage of positive tubules in *Trail* ^−/−^ mice dropped to 6.08% while wild type mice of the same age maintained the same proportion of CC8 positive tubules (2.83%).

The activation of the intrinsic apoptotic signaling pathway was assessed through detection of the cleaved form of caspase 9 (CC9, Fig. 6B). Interestingly, *Trail* ^−/−^ mice also had more CC9 positive tubules than wild type mice at PND 28 and PND 56. At PND 28, *Trail* ^−/−^ mice have a basal level of CC9 positive tubules (14.70%) that is 7 fold higher than age-matched C57BL/6J mice (2.03%). By PND 56, the number of CC9 positive tubules in the *Trail* ^−/−^ mice decreased slightly (10.45%) while C57BL/6J mice remained at a low level of CC9 activity (3.93%). Taken together, the observed increases in both CC8 and CC9 in *Trail* ^−/−^ mice indicate that the *Trail* ^−/−^ mice have a more robust basal activity of both the extrinsic and intrinsic apoptotic pathways, which together underlies the mechanism accounting for the increased apoptotic index observed in *Trail* ^−/−^ mice at both ages.

## Discussion

The data in this manuscript show an increase in the incidence of germ cell apoptosis in TRAIL gene deficient mice and that the loss of TRAIL disrupts the first wave of spermatogenesis as well as later cycles. *Trail^−/−^* mice in this study carry a partial deletion of exon 2 and a complete deletion of exon 3 in the *Trail* gene [23]. It has been reported that a HeLa cell line transfected with two alternative splicing transcripts of TRAIL (TRAIL-β and TRAIL-γ) has a decrease in DNA fragmentation and cell death. This cell line harbors both TRAIL-β and TRAIL-γ, resulting in a truncated TRAIL protein lacking the extracellular domain [24]. Conversely, our results show an increase of germ cell apoptosis in pubertal (PND 28) and adult (PND 56) *Trail^−/−^* mice compared to C57BL/6J of the same age (Fig. 2E). This increase in germ cell apoptosis may result from compensation by another death receptor signaling family member, such as FasL, that is known to participate in the instigation of germ cell apoptosis in the testis [20], or a possible compensatory activity of the intrinsic apoptotic pathway in order to maintain homeostasis during testis development (Fig. 6). Studies in hepatocytes (primary hepatocytes and HepG2) show that TRAIL contributes to enhanced intrinsic pathway signaling and subsequent apoptosis [25], [26]. When TRAIL fails to trigger apoptosis in these cells, the MAP kinase, JNK, can still be activated [25]. In this study, our results indicate that the loss of TRAIL signaling leads to the activation of the intrinsic apoptotic pathway in the testis (Fig. 6B).

TRAIL is known to induce apoptosis in transformed cells, yet soluble TRAIL generally has little pro-apoptotic activity in non-transformed cells. However, our laboratory has reported that the addition of TRAIL and an anti-DR5 monoclonal antibody MD5-1 can induce a synergistic increase of apoptosis in p53 permissive GC-2spd (ts) cells [19]. Although TRAIL is expected to induce apoptosis in a majority in tumor cells with less of an effect in the most normal cells and tissues [7], its function in normal, non-transformed tissues is not clear. Several reports show that TRAIL has the ability to active mitogen-activated protein (MAP) kinase, nuclear factor (NF)-κB, and protein kinase B (PKB or Akt); which are all involved in pro-survival or non-apoptotic signaling [27], [28], [29]. Recent studies have suggested that suppressing apoptosis with a caspase inhibitor can enhance TRAIL’s stimulation of the NF-κB pathway [29]. Also, it has been observed that TRAIL-R can enhance cholangiocarcinoma metastasis by activation of the NF-κB signaling pathway [30]. It is possible that the loss of TRAIL expression in the testis causes an elevation of apoptosis when TRAIL cannot trigger these non-apoptotic pathways.

In addition to examining the function of TRAIL in normal testis development and spermatogenesis, we evaluated the testis of *Trail^−/−^* mice during the pubertal period (PND 28) and adulthood (PND 56). C57BL/6J mice had a higher testis to body weight ratio at PND 28 and PND 56 (Table 1). No significant differences were observed in the body weight of C57BL/6J and *Trail^−/−^* mice at either age group, but *Trail^−/−^* mice showed a significant decrease in testis weight at both PND 28 and PND 56 (Table 1). *Bclw,* which is a member of the *Bcl2* gene family, is an anti-apoptotic protein involved in apoptosis during development of the testis. A similar decrease in testis weight was found in Bclw^−/−^ mice [31]. These findings suggest that the loss of a pro-apoptotic or anti-apoptotic protein may lead to compensation by the extrinsic and intrinsic apoptotic pathways following a disruption of homeostasis during development of the testis.

In order to determine whether the loss of TRAIL can alter reproductive capacity, the number of mature spermatid heads was evaluated in C57BL/6J and *Trail^−/−^* mice at PND 56. A reduction in mature spermatid head counts was found in *Trail^−/−^* mice compared to C57BL/6J mice (Fig. 1). The low value observed at PND 56 may be due to the continued high germ cell apoptotic frequency at PND 28 (Fig. 2). It is possible that the reduced number of spermatid heads results from altered meiosis during spermatogenesis at the pubertal age, causing the lower spermatid head counts in *Trail^−/−^* mice observed in the adult (Fig. 3). The delayed spermatogenesis in *Trail^−/−^* mice at PND 56 was not a prolonged effect, yet the *Trail^−/−^* mice still have more meiotic germ cells than wild type mice. Mice deficient in *Bclw* or *Bax* often suffer a loss of fertility [31], [32], but the decreased number of spermatid heads did not dramatically influence the reproductive capacity of *Trail^−/−^* mice. Although the reduction of spermatid heads of *Trail^−/−^* mice does not significantly alter the ability of these mice to reproduce, the time to litters is longer than in C57BL/6J mice (data not shown).

Itch, a known E3 ligase, targets proteins in immune system, including cFLIP, the anti-apoptotic protein cellular FLICE-like inhibitory protein. Itch can degrade cFLIP to prevent its inhibition of caspase-8 [33]. The high basal apoptotic rates in the testis were also observed in itchy*^−/−^* mice, which are similar to *Trail^−/−^* mice at both pubertal and adult ages [34]. An important difference is that *FasL^−/−^* mice only show high basal apoptosis at the pubertal age, not in adult [20]. Recent reports indicate that Fas regulates spermatocyte apoptosis in rats during the first wave of spermatogenesis as a mechanism to establish an appropriate population size [21]. This suggests that TRAIL and ITCH may play more dominant roles than FasL in controlling the population of germ cells after the first wave of spermatogenesis, while FasL may be more critical during the pubertal period [20].

Histological analysis of the adult *Trail^−/−^* mice revealed some alterations in the seminiferous epithelium. At PND 28, *Trail^−/−^* mice had more tubules with meiotic figures and less tubules with late stage spermatids compared to C57BL/6J mice, suggesting that the loss of TRAIL can influence cell division (Fig. 3–4). A similar phenomenon is also found in the *Itcy^−/−^* mice, though a detailed mechanism is still unclear [34]. If the increase in meiotic spermatocytes is due to a delay or arrest of maturation, the progression of normal spermatogenesis will be altered. At PND 56, the number of meiotic figures decreased to 8.13% in the *Trail^−/−^* mice, but this proportion is still greater than that observed in C57BL/6J mice (1.8 fold). Although adult *Trail^−/−^* mice can still produce mature and functional spermatozoa, the number of sperm is less than C57BL/6J. This suggests that the meiotic delay may lengthen spermatogenesis, causing fewer mature spermatids form. In addition, the high apoptotic rate and low testis weight in PND 56 *Trail^−/−^* mice may be influenced by this meiotic delay.

Taken together, the results presented in this study provide novel evidence that TRAIL is important in regulating germ cell apoptosis during the first wave of spermatogenesis, and in maintaining testicular germ cell homeostasis at later ages.

## Materials and Methods

### Ethics Statement

All procedures involving mice were performed in accordance with the guidelines of the University of Texas at Austin’s Institutional Animal Care and Use Committee (IACUC) in compliance with guidelines established by the National Institutes of Health. The animal experiments described in this manuscript were specifically approved by the IACUC (approval # AUP-2012-00046).

### Animals

All mice used in the experiments were maintained at The University of Texas at Austin’s Animal Resource Center and were housed at a constant temperature (22±0.5°C) at 35–70% humidity with a 12L:12D photoperiod. Mice were given standard lab chow and water *ad libitum*. Breeding pairs of wild-type C57BL/6J mice were purchased from The Jackson Laboratory (Bar Harbor, ME). Breeding pairs of *Trail* gene deficient (*Trail^−/−^*) mice were provided by Amgen Inc. (Thousand Oaks, CA) [35]. Two ages of mice were selected for this study; postnatal day (PND) 28 representing the pubertal period and adult PND 56. The pubertal age was selected since it is known that an increased incidence of germ cell apoptosis occurs during this developmental period [3]. Mice were killed at postnatal day PND 28 and PND 56 by CO_2_ inhalation followed by cervical dislocation, and the body and testis weight were recorded. The testes were rapidly removed and either frozen in liquid nitrogen and stored at −80°C for protein analysis, or immersion-fixed overnight in Bouin’s solution (Polysciences, Inc., Warrington, PA), washed in 70% ethyl alcohol-Li_2_CO_3_ saturated solution and embedded in paraffin for histology analysis.

### Genotyping PCR and Primers

Wild type C57BL/6J and *Trail^−/−^* mice were confirmed using genotyping PCR with primers specific for the *Trail* gene [35]. The mutant TRAIL allele was detected by PCR analysis of tail lysis genomic DNA, using primers 5-AAA GAC GGA TGA GGA TTT CTG GG-3′ and 5′-GAC AGA ACA CCA TAT TGC TGG CG-3′ specific to the TRAIL/Apo2L sequence, and primers 5′-GCC CTG AAT GAA CTG CAG GAC G-3′ and 5′-CAC GGG TAG CCA ACG CTA TGT C-3′ specific to neomycin sequence. Genotyping PCR conditions were performed in 35 cycles of 94°C for 1 minute, 66°C for 1 minute, 72°C for 40 seconds, and 72°C for 5 minutes. The wild type primer results in 240 bp single band, while the mutant primer results in 520 bp single band (data not shown).

### Physiological Characterization

In order to characterize the basic testicular phenotype in *Trail^−/−^* mice during development, pubertal (PND 28) and adult (PND 56) were selected. The testes weights were calculated for each mouse, and represented as an average of the left testes and the right testes. The testis/body weight ratio is computed by dividing the average testes weight by body weight. A minimum number of 18 mice were used for each genotype in each group.

### Testicular Spermatid Head Counts

Testicular spermatid head counts in PND 56 mice were performed as previously described with slight modifications [20], [36]. Briefly, testes were homogenized in a solution containing 0.9% (w/v) sodium chloride (NaCl) and 10% (v/v) dimethyl sulfoxide (DMSO). Homogenization-resistant spermatid heads were counted on a hemocytometer by using a Nikon E800 microscope (Nikon Instrument Inc., Melville, NY). The average number of spermatid heads were determined from 9 C57BL/6J mouse testes and 8 TRAIL^−/−^ mouse testes. Each testis sample was counted 3 times. The daily sperm production per testis was calculated by using 4.84 days as the time divisor [37].

### Terminal Deoxy-nucleotidyl Transferase-mediated Digoxigenin-dUTP Nick End Labeling (TUNEL) Assay

Apoptotic fragmentation of DNA in mouse paraffin-embedded testis cross sections was determined by TUNEL analysis using the ApopTag™ kit (EMD Millipore, Billerica, MA). The apoptotic index (AI) was calculated as the percentage of essentially round seminiferous tubules in a cross section that contained more than 3 TUNEL-positive germ cells [20], [38]. At least 2 testicular cross sections per mouse were analyzed, and at least 11 mice were counted from each age group (PND 28: C57BL/6J mice (n = 10) and *Trail^−/−^* mice (n = 13); PND 56: C57BL/6J mice (n = 11) and 14 *Trail^−/−^* mice (n = 14)).

### Testicular Histology and Meiotic Quantification

Cross sections (5 µm) of paraffin-embedded testes were evaluated for morphological changes by using Periodic Acid-Schiffs-Hematoxylin (PAS-H) staining. All slides were viewed on Nikon E800 microscope and images were captured using a Nikon digital DS camera (Nikon Instrument Inc., Melville, NY) or Aperio ScanScope system (Aperio Technologies, Vista, CA). The method for assessing meiotic figures is based upon criteria as detailed in *Histological and Histopathological Evaluation of the Testis (LD Russell et al)* and modified by Dr. Yokonoshi [39] and by Dr. Dwyer in one of our previous publications [34]. In short, due to a lack of elongated spermatids in young animals, it is difficult to precisely identify tubule stage. Some tubules appear to be at Stage 12, the only stage would be expected to see meiosis normally, but in fact there are more than three cell types present, suggesting these tubules are more likely in Stage 10 to Stage 2/3. Therefore, if a tubule contained cells that were undergoing meiosis, we labeled that tubule as having meiotic figures, rather than stating it was a Stage 12 tubule. The number of mice by type used for the evaluation of meiotic quantification: PND 28 C57BL/6J mice (n = 12) and TRAIL^−/−^ mice (n = 14); PND 56 C57BL/6J mice (n = 12) and TRAIL^−/−^ mice (n = 15).

### Total Protein Extraction and Western Blot Analysis

As detailed description of total protein preparation from mouse testis and Western blot analysis have been described previously [38]. Briefly, total protein was collected from 2 sets of whole testes homogenized in RIPA buffer and the concentration was determined by using the Bio Rad DC Protein Assay (Life science, Hercules, CA, #500-0111). For each sample, 30 µg total protein was separated using a 4–12% NuPAGE gradient gel (Life Technologies, Grand Island, NY), transferred to a PVDF membrane (Life Technologies, Grand Island, NY), and blocked using a 5% milk solution. Total cellular proteins were detected using primary antibodies against FasL (Santa Cruz biotechnology, Inc., Dallas, TX, sc956, 1∶2,000) and α-Tubulin (Cell Signaling Technology Inc., Danvers, MA, #2144, 1∶5,000) coupled with horseradish-conjugated secondary antibody (Cell Signaling Technology Inc., Danvers, MA, #7074, 1∶5000). The ECL chemiluminescent substrate (GE Healthcare bio-science, Pittsburg, PA) was used as the detection reagent and α-tubulin as the internal control for gel loading. All experiments were performed in triplicate and repeated at least three times.

### Testicular Immunohistochemistry

Expression of cleaved caspase-8 and cleaved caspase-9 in the germ cells was determined by immunohistochemistry. Cross sections (5 µm) of paraffin-embedded testes from PND 28 C57BL/6J and TRAIL^−/−^ mice were deparaffinized and rehydrated, and antigens were unmasked by heating in 10 mM sodium citrate solution. Sections were incubated with 3% H_2_O_2_ to block endogenous peroxidase activity and then incubated in blocking buffer containing 10% horse serum. The primary antibodies used were cleaved caspase-8 (Cell Signaling Technology Inc., Danvers, MA, #8592, 1∶200) and cleaved caspase-9 (Cell Signaling Technology Inc., Danvers, MA, #9509, 1∶200). Sections were incubated in primary antibody at 4°C overnight. Immunodetection was performed by standard procedures using the Vecta Stain ABC kit (Vector Labs, Burlingame, CA) and DAB as the substrate (Vector Labs). At least 2 testicular cross sections per mouse were analyzed. The number of mice by type used for the evaluation of immunohistochemistry: Cleaved-caspase-9: PND 28 C57BL/6J mice (n = 12) and TRAIL^−/−^ mice (n = 12); PND 56 C57BL/6J mice (n = 10) and TRAIL^−/−^ mice (n = 12). Cleaved-caspase-8: PND 28 C57BL/6J mice (n = 13) and TRAIL^−/−^ mice (n = 10); PND 56 C57BL/6J mice (n = 5) and TRAIL^−/−^ mice (n = 11). All slides were viewed on Nikon E800 microscope and images were captured using a Nikon digital DS camera (Nikon Instrument Inc., Melville, NY) or Aperio Scan Scope system (Aperio Technologies, Vista, CA). The index was expressed as the percentage of essentially round tubules that contained more than 3 cleaved caspase-8 or cleaved clapase-9 germ cells.

### Statistical Analysis

In this study, the minimum number of animals necessary to achieve statistical significance was determined by statistical power analysis (α  = 0.05, β  = 0.05) [40], [41]. Statistical analysis was performed using Prism 5 (Graph Pad Software, Inc., La Jolla, CA). Statistical results were presented as the individual means ± SEM. The data were subjected to a Student’s *t*-test. Comparisons were considered statistically significant when *p*<0.05.
